# Occult cytomegalovirus infection presents anastomotic leakage after gastrectomy: Two case reports

**DOI:** 10.1097/MD.0000000000047254

**Published:** 2026-01-16

**Authors:** Jun Hyung Kim, Il Jo, Sungho Lee, Kwanhoon Park, Kang Yoon Lee, Da Hyun Jung, Han-Gil Yoon, Ji Young Jang

**Affiliations:** a Department of Surgery, National Health Insurance Service Ilsan Hospital, Goyang, Gyeonggi-do, Republic of Korea; b Nursing Education Support Department, National Health Insurance Service Ilsan Hospital, Goyang, Gyeonggi-do, Republic of Korea.

**Keywords:** anastomotic leak, cytomegalovirus infections, gastrectomy

## Abstract

**Rationale:**

Cytomegalovirus (CMV) remains latent and causes disease in immunocompromised patients. However, surgical stress may also trigger reactivation without overt immunosuppression, leading to severe complications.

**Patient Concerns:**

Both patients developed life-threatening postoperative bleeding and anastomotic leakage after gastrectomy. The complications were difficult to resolve, leading to transfer to our hospital’s intensive care unit in the first case and repeated reoperations for leakage in the second case.

**Diagnoses:**

Refractory postoperative anastomotic leakage associated with occult CMV infection.

**Interventions:**

CMV infection was confirmed by whole-blood CMV PCR and treated with intravenous ganciclovir. Supportive measures, including endoscopic vacuum therapy, were performed to manage the anastomotic defects.

**Outcomes:**

In Case 1, leakage and bleeding persisted despite antiviral therapy, resulting in death from respiratory failure. In Case 2, antiviral initiation following CMV diagnosis led to significant improvement in anastomotic healing, and recovery without further major complications.

**Lessons:**

Occult CMV infection should be considered in patients with recurrent postoperative anastomotic leakage, even in the absence of overt immunosuppression. Early diagnosis and prompt antiviral-therapy may improve outcomes.

## 1. Introduction

Cytomegalovirus (CMV), a β-herpesvirus with global seroprevalence 40–100%, establishes lifelong latency in myeloid progenitor cells.^[1]^ Transmission occurs through body fluids, including sexual contact, and during the perinatal period.^[2]^ Typically, CMV infection occurs in fetuses or immunocompromised individuals, such as those undergoing chemotherapy, organ transplantation, or immunosuppressive therapy.^[3,4]^ While reactivation is classically associated with profound immunosuppression, CMV disease may also develop in patients without overt immunodeficiency, particularly in the presence of physiological stress, malignancy, or major surgery.^[2]^ In such cases, CMV reactivation may manifest as postoperative complications, such as anastomotic leakage or bleeding, potentially obscuring diagnostic clues and delaying appropriate antiviral treatment, sometimes with fatal consequences.

We present 2 postoperative cases in which a delayed diagnosis of CMV contributed to persistent anastomotic leakage and severe postoperative clinical deterioration. The first case involved an 84-year-old woman who underwent a total gastrectomy for a bleeding perforated ulcer at the gastric cardia. The patient subsequently developed an esophagojejunal anastomotic leak, which significantly impeded her recovery. CMV esophagitis was later confirmed histologically, and earlier recognition may have altered the patient’s clinical course. The second case involved a 67-year-old man who underwent a laparoscopic subtotal gastrectomy for advanced gastric cancer. The patient developed recurrent anastomotic leakage despite multiple surgical revisions and endoscopic vacuum therapy (EVT). CMV viremia was eventually detected and considered a potential contributing factor. These cases underscore the importance of considering CMV infections in patients with unexplained or refractory postoperative anastomotic complications.

## 2. Case reports

### 2.1. Case 1

An 84-year-old woman with hypertension, chronic kidney disease, hypothyroidism, and Parkinson disease was admitted to a tertiary hospital with acute cerebral infarction involving the right cerebellum. According to her family, she experienced a rapid decline in physical function approximately 3 months prior to admission following the death of her spouse. During hospitalization, inflammatory markers were elevated and abdominopelvic computed tomography (APCT) revealed gastric perforation around the cardia. The patient underwent emergency total gastrectomy with Roux-en-Y esophagojejunostomy (EJ). Operative findings included a 6-cm deep ulcer with arterial spurting bleeding at the cardia, severe inflammation extending up to the esophagogastric junction, and microperforation near the upper margin of the ulcer. Melena persisted despite surgical intervention. Her vital signs became increasingly unstable and she was transferred to our hospital for critical care on postoperative day (POD) 29.

On March 23, 2024, upon arrival at our hospital, the patient required norepinephrine at a rate of 0.15 μg/kg/min to maintain hemodynamic stability and was receiving supplemental oxygen via a reservoir mask at 5 L/min. Within the first 6 hours of admission, she passed approximately 500 cc of melena and had a urine output of only 15 cc. The patient was promptly admitted to the intensive care unit (ICU), where endotracheal intubation and continuous renal replacement therapy were initiated because of hemodynamic instability and altered mental status.

**Figure 1.**
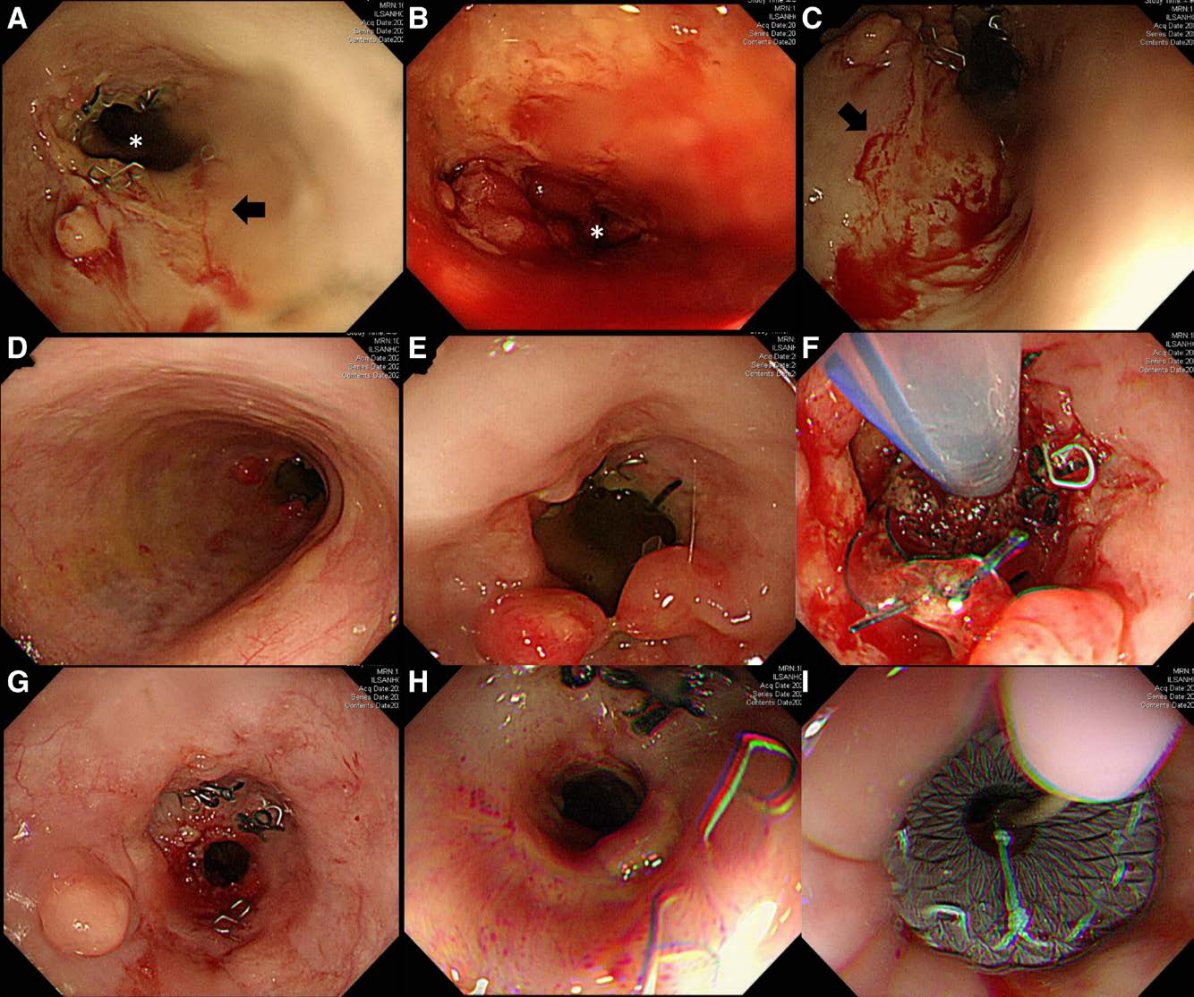
Endoscopy findings in Case 1. (A, B, C) Following total gastrectomy with esophagojejunostomy, EGD revealed multiple extensive esophageal ulcers with oozing both above (black arrow) and below (white asterisk) the anastomosis site. Injection sclerotherapy was performed for the oozing lesions. (D, E) Follow-up EGD on hospital day 16 showed ulcer improvement, and (F) an intraluminal EVT was performed. (G) The image on hospital day 37 shows anastomosis stenosis prior to balloon dilatation, captured after EVT removal. (H) EGD on hospital day 54 shows post-dilatation improvement. (I) A SEMS was inserted.

**Figure 2.**
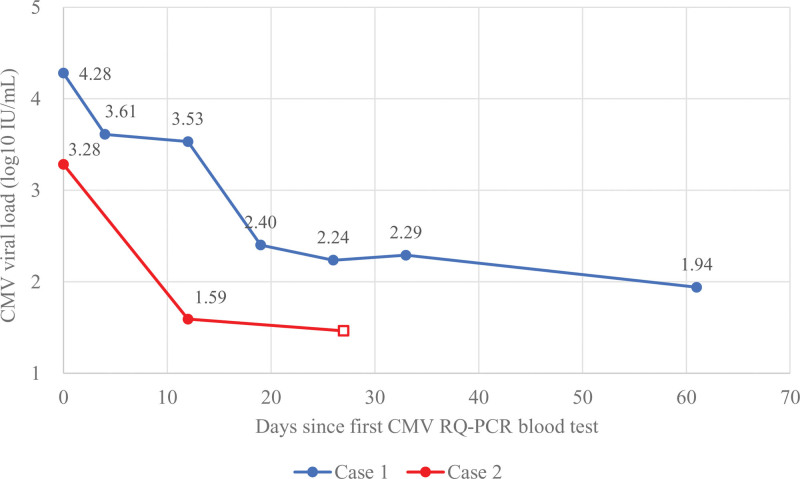
CMV viral-load trajectories (log10 IU/mL) plotted by days since the first CMV RQ-PCR blood test (Day 0). CMV viral-load trajectories (log10 IU/mL) by days since the first CMV RQ-PCR blood test (Day 0). Lines depict Case 1 and Case 2. An open square denotes a value below the assay lower limit of detection (LOD < 30 IU/mL) and is plotted at log10(30) = 1.48 for visualization only. “Days since first CMV RQ-PCR blood test” is defined separately for each case as the number of days from the index test (Day 0).

On hospital day (HOD) 2 after stabilization of her vital signs, esophagogastroduodenoscopy (EGD) was performed and revealed multiple oozing ulcers in the esophagus and jejunum (Figs. 1A-1C). Hemostasis was attempted using an epinephrine injection. Although stress-related or drug-induced ulcers were initially suspected, the atypical and extensive distribution of the lesions, particularly around the cardia and anastomotic site, raised concern for alternative etiologies such as viral infections or malignancy. However, a biopsy could not be performed because of tissue fragility and thrombocytopenia (platelet count: 33,000/µL), as per the endoscopist’s recommendation. Subsequently, blood CMV real-time quantitative polymerase chain reaction (RQ-PCR) revealed a significant viral load of 19,100 IU/mL, prompting the initiation of intravenous ganciclovir therapy (Fig. 2 and Table S1, Supplemental Digital Content, https://links.lww.com/MD/R167). Review of an external slide of the total gastrectomy specimen from the previous hospital showed no evidence of CMV or malignancy.

EGD = esophagogastroduodenoscopy, EVT = endoscopic vacuum therapy, SEMS = self-expandable metal stent.

CMV = cytomegalovirus, RQ-PCR = real-time quantitative polymerase chain reaction.

APCT performed on HOD 3 revealed dehiscence of the esophagojejunal anastomosis and active bleeding at the proximal jejunum mid-abdomen, unrelated to the anastomosis. To maintain the hemoglobin level at approximately 7.0 g/dL, daily transfusions of 2 units of packed red blood cells were required, suggesting ongoing blood loss. Angioembolization of the jejunal branch was performed on HOD 4. On HOD 6, the patient’s condition improved with an increase in urine output, which enabled transition from continuous renal replacement therapy to conventional intermittent hemodialysis. On HOD 16, follow-up serology results showed a decreased CMV titer and endoscopy revealed improved ulcers; thus, an EVT was initiated in consultation with the endoscopist (Fig. 1D–F). Initially, owing to the bleeding tendency, continuous negative pressure was applied at 60 mm Hg, which was later increased to 100 mm Hg. The patient was extubated on HOD 18.

After consulting an upper gastrointestinal surgeon, surgical treatment for the anastomotic leakage was deemed unfeasible because of the patient’s advanced age, overall condition, and underlying comorbidities. Consequently, conservative and endoscopic treatments were recommended. On HOD 32, follow-up EGD confirmed the continued need for EVT therapy, with plans for its removal at the next follow-up. On HOD 37, another EGD revealed significant stenosis at the esophagojejunal junction, which resulted in urgent EVT removal and balloon dilation, with plans to consider stent insertion within 2 weeks (Fig. 1G). The patient’s condition improved, and she was transferred from the ICU to the general ward on HOD 41. Antiplatelet therapy was restarted after transfer, having been withheld in the ICU due to persistent bleeding tendency. On HOD 54, a 6-cm antireflux-covered self-expandable metal stent was inserted endoscopically (Figs. 1H, 1I). The patient was kept under observation and received enteral nutrition via a nasogastric tube. Although she initially appeared to stabilize under supportive care, her condition abruptly deteriorated following an aspiration event, which led to acute hypoxic respiratory failure and subsequent cardiac arrest. Despite resuscitation efforts, she died on July 16, 2024.

### 2.2. Case 2

A 67-year-old man with a history of open low anterior resection followed by adjuvant FOLFOX chemotherapy for sigmoid adenocarcinoma was diagnosed with moderately differentiated adenocarcinoma in the gastric antrum during a routine 7-year surveillance EGD. The patient had no documented comorbidities, and baseline laboratory findings were unremarkable. On February, 24, 2025, the patient underwent laparoscopic subtotal gastrectomy with Roux-en-Y gastrojejunostomy (GJ) and D2 lymphadenectomy. The surgery was uneventful, with mechanical anastomosis performed, and no intraoperative transfusion or coagulation defect was observed. The final pathological examination confirmed pT3 N3a, R0 resection.

**Figure 3.**
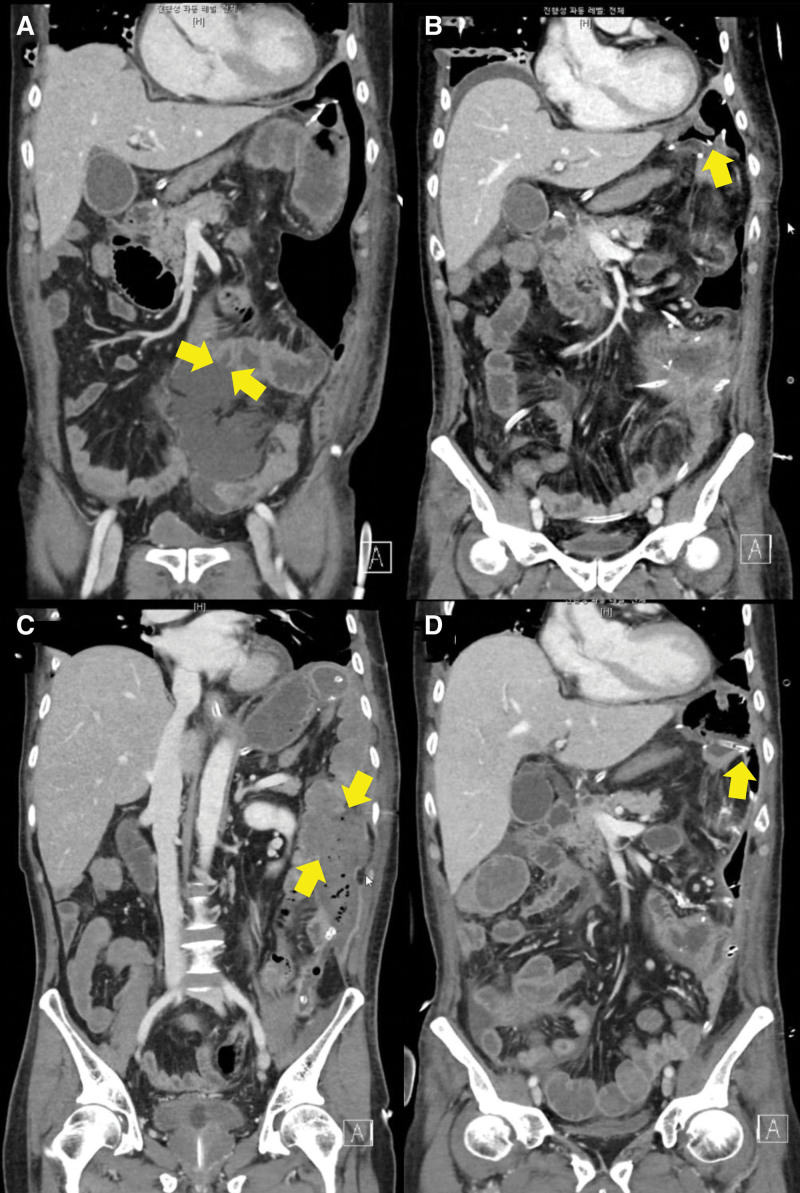
Serial abdominal CT images demonstrating progressive gastrointestinal complications in Case 2. (A) Coronal CT image on March 5, 2025 shows a 1-cm wall defect in the mid-descending colon with adjacent fluid collection in the left abdomen (yellow arrows), and an additional 0.8-cm wall defect abutting the small bowel near the jejunojejunostomy site. (B) On March 7, 2025, a small defect is noted at the gastrojejunal anastomosis, accompanied by peri-gastric free air (yellow arrow). (C) CT on March 22, 2025 reveals interval progression with a large (4.2 cm) wall defect of the mid-descending colon and a dehisced (approximately 4 cm) gastrojejunal anastomosis (yellow arrows). (D) Follow-up CT, on March 30, 2025, shows probable redevelopment of a approximately 4 cm defect at the gastrojejunostomy site (yellow arrow), and a questionable small dehiscence at the previously repaired descending colon.

On POD 9, routine laboratory tests revealed an acute decline in hemoglobin from 7.4 to 6.4 g/dL. APCT revealed pneumoperitoneum and 2 discrete wall defects: a 1-cm perforation in the mid-descending colon and a 0.8-cm defect along the anti-mesenteric border of the jejunojejunostomy site (Fig. 3A). Emergency exploratory laparotomy confirmed the presence of localized peritonitis. Both perforations were primarily repaired. On POD 2 after reoperation, a bilious fluid was observed in the left Jackson-Pratt drain. Follow-up CT revealed a small defect in the gastrojejunal anastomosis (Fig. 3B). EGD confirmed gastrojejunal anastomotic dehiscence and EVT was subsequently initiated. Following a period of clinical stabilization, the patient experienced sudden deterioration on March 22 with an abrupt discharge of old blood and profuse bowel contents through the surgical wound. Emergency contrast-enhanced CT revealed a 4-cm defect at the site of the previous descending colon repair and a 3-cm dehiscence at the gastrojejunal anastomosis (Fig. 3C). A re-exploratory laparotomy was performed, during which the recurrent colonic perforation was repaired and an ascending loop colostomy was constructed for diversion. The GJ defect was primarily repaired.

CT = computed tomography.

On POD 7, sudden discharge of approximately 800 mL of old bloody fluid from the colostomy was observed, raising concerns about ongoing gastrointestinal bleeding. Contrast-enhanced CT did not reveal active extravasation; however, a 4-cm wall defect was again noted at the gastrojejunal anastomosis, suggesting recurrent dehiscence. A small defect was suspected at the site of the previously repaired mid-descending colon (Fig. 3D).

Given the recurrent anastomotic complications, residual gastric malignancy and Helicobacter-associated ulcer were considered, but repeat endoscopic biopsy was negative. In the colon, *Clostridioides difficile* infection related to prior antibiotic exposure was suspected, but testing was also negative. During the prolonged ICU stay, the patient received anticoagulant therapy for deep vein thrombosis prophylaxis due to immobilization. However, no anticoagulants were used during the acute phase, and no other bleeding- or ulcer-inducing medications were administered. Because of the repeated and multifocal lesions, a systemic cause was considered. In particular, the unusually frequent anastomotic dehiscence raised suspicion of CMV infection as the underlying etiology. On POD 9, although immunohistochemical staining for CMV performed on the archived tissue slide from the initial gastrectomy specimen was negative, blood CMV RQ-PCR returned a positive result of 1923 IU/mL, and empiric intravenous ganciclovir therapy was promptly initiated (Fig. 2 and Table S1, Supplemental Digital Content, https://links.lww.com/MD/R167). On POD 22, the PCR levels decreased to 39 IU/mL, and endoscopic evaluation during EVT session 5 demonstrated marked improvement in the anastomotic defect, with biopsy specimens showing negative results for both CMV and malignancy. The EVT device was subsequently removed and the patient recovered with conservative management, including gradual dietary advancement and close clinical monitoring, without evidence of further bleeding or recurrent anastomotic leakage. He was eventually discharged on hospital day 178 without evidence of further bleeding or recurrent anastomotic leakage.

The ganciclovir dosing schemes^[5,6]^ and timelines for both cases are summarized in Tables S2 and S3, Supplemental Digital Content, https://links.lww.com/MD/R167.

## 3. Discussion

Given the increasing number of major surgeries performed on aging populations, particularly in regions with high CMV seroprevalence, awareness of latent CMV reactivation in surgical patients has important clinical implications. CMV is highly prevalent in many developing countries and similarly elevated seroprevalence rates have been reported in industrialized Asian countries.^[7,8]^ For example, maternal CMV seropositivity has been reported to be 82.5% in Japan and 97% in Korea.^[9,10]^ In such regions, widespread latent infections may contribute to an increased susceptibility in certain clinical settings, underscoring the need for increased vigilance in the recognition of active infections.

### 3.1. Clues to CMV infection

The first patient’s general condition deteriorated significantly after the death of her spouse in 2023. She had been receiving antithrombotic therapy for a prior cerebral infarction and presented with a gastric ulcer located near the cardia, corresponding to a modified Johnson type IV lesion,^[11,12]^ which was an atypical site for peptic ulcers, raising suspicion of a stress-related or drug-induced etiology.^[13]^ In addition, EGD revealed multiple extensive esophageal ulcers oozing both above and below the anastomosis (Figs. 1A-1C), strongly suggestive of viral esophagitis, particularly CMV infection. Similar endoscopic findings were also observed in Case 2, showing discrete ulcerative lesions with exudate around the anastomotic site, a characteristic feature of CMV infection, providing additional visual clues suggestive of CMV infection (Figure S1, Supplemental Digital Content, https://links.lww.com/MD/R167).^[14]^

In the second case, the patient had been evaluated by a nutritionist before gastric cancer surgery and was considered nutritionally adequate.^[15]^ Although the patient appeared clinically immunocompetent, he had previously received adjuvant chemotherapy and undergone 2 major abdominal surgeries under general anesthesia. These factors may have contributed to the transient immunosuppression, rendering CMV reactivation plausible. However, the infection remained clinically silent and was suspected only after multiple episodes of critical hemorrhage or perforation.

These cases illustrate that CMV infection can be easily overlooked, particularly when not actively considered in the differential diagnosis of postoperative complications. In surgical practice, we occasionally encounter patients whose postoperative course is marked by unusually persistent or recurrent complications, despite appropriate management. Recognizing this pattern should prompt clinicians to consider CMV reactivation as a potential underlying cause. While histopathology remains the gold standard for CMV diagnosis, its limited sensitivity and delayed results may hinder the timely initiation of appropriate treatment. In these cases, diagnosis was established by CMV PCR testing of whole blood, and supported by compatible clinical features. Given its superior sensitivity and faster turnaround time, PCR may serve as a practical first-line diagnostic tool for postoperative patients with unexplained or refractory complications.

### 3.2. Effect of antiviral treatment

Early diagnosis of CMV infection was associated with reduced in-hospital mortality and improved overall survival.^[16]^ In emergency gastrointestinal surgery patients diagnosed with CMV infection, a median time of 9 days was reported from surgery to the initiation of ganciclovir treatment, with a reoperation rate of 22.2% and in-hospital mortality of 25.9%.^[17]^

In all cases, CMV infection was not suspected until severe anastomotic or colonic complications developed. Following the initiation of intravenous ganciclovir therapy, the CMV viral loads measured by quantitative PCR declined dramatically in both cases, and the patients’ clinical conditions subsequently stabilized (Fig. 2 and Table S1, Supplemental Digital Content, https://links.lww.com/MD/R167). If a CMV infection had been suspected earlier, timely PCR testing and prompt antiviral therapy might have mitigated the progression of these complications. These findings suggest that early CMV PCR testing and empirical initiation of antiviral therapy should be strongly considered in patients with unexplained or refractory postoperative complications.

## 4. Conclusion

CMV infection is frequently underdiagnosed in postoperative patients without clearly defined immunosuppressive conditions, particularly in regions with high seroprevalence, including both developing and industrialized countries. The potential for life-threatening complications underscores the need for increased clinical suspicion, especially in patients presenting with unexplained or recurrent postoperative deterioration. To support earlier recognition, further research is warranted to define the indications and clinical utility of expanded diagnostic screening strategies. In selected cases, empirical administration of ganciclovir may be justified even in the absence of histopathological confirmation, particularly when clinical features and laboratory data strongly suggest CMV reactivation.

## Acknowledgments

We thank the patients and their families for allowing us to report these cases. Artificial intelligence tools (ChatGPT by OpenAI) have been used to refine the language and grammar of this manuscript. No AI tools were used for data analysis or interpretation. All final decisions and contents are the responsibility of the authors.

## Author contributions

**Conceptualization:** Jun Hyung Kim, Il Jo, Ji Young Jang.

**Data curation:** Jun Hyung Kim, Il Jo.

**Investigation:** Jun Hyung Kim, Il Jo.

**Methodology:** Jun Hyung Kim, Il Jo.

**Resources:** Sungho Lee, Kwanhoon Park, Kang Yoon Lee, Da Hyun Jung, Han-Gil Yoon.

**Supervision:** Ji Young Jang.

**Validation:** Sungho Lee, Ji Young Jang.

**Visualization:** Jun Hyung Kim, Il Jo.

**Writing – original draft:** Jun Hyung Kim, Il Jo, Kwanhoon Park, Kang Yoon Lee, Da Hyun Jung, Han-Gil Yoon, Ji Young Jang.

**Writing – review & editing:** Jun Hyung Kim, Il Jo, Sungho Lee.

## Supplementary Material


