# Supplementary material for: Occult cytomegalovirus infection presents anastomotic leakage after gastrectomy: Two case reports
Source: Medicine (Baltimore). 2026 Jan 16;105(3):e47254. doi: 10.1097/MD.0000000000047254 (PMC12826198; doi:10.1097/MD.0000000000047254)

Supplementary Table S1. CMV RQ-PCR blood levels by days since the first CMV RQ-PCR blood test.

Day	CMV RQ-PCR, Blood (IU/mL)
Case 1	
0	19,100
4	4,083
12	3,406
19	252
26	172
33	195
61	87
Case 2	
0	1,923
12	39
27	<30[†]

Day 0 is defined for each case as the date of the first CMV RQ-PCR blood test.

[†] Values below the assay lower limit of detection (LOD <30 IU/mL) were denoted accordingly.

Supplementary Table S2. Ganciclovir dosing schema.

Ganciclovir dose	
Induction dose	5 mg/kg IV q12h for 14 days<br>Dose in renal impairment<br>CrCl ≥70: No dosage adjustment<br>CrCl 50–69: 2.5 mg/kg q12h<br>CrCl 25–49: 2.5 mg/kg q24h<br>CrCl 10–24: 1.25 mg/kg q24h<br>CrCl <10: 1.25 mg/kg 3x/week<br>Hemodialysis<br>1.25 mg/kg 3x/week (dose after dialysis on dialysis days)<br>CRRT<br>2.5 mg/kg q24h
Maintenance dose	5 mg/kg IV q24h<br>Dose in renal impairment<br>CrCl ≥90: No dosage adjustment<br>CrCl 50–89: 2.5–5 mg/kg q24h<br>CrCl 10–50: 0.625–1.25 mg/kg q24h<br>CrCl <10: 0.625 mg/kg 3×/week<br>Hemodialysis<br>0.625 mg/kg 3×/week (dose after dialysis on dialysis days)

CrCl, creatine clearance (mL/min) estimated by the Cockcroft–Gault equation.

Supplementary Table S3. Timeline of two cases

Case 1		
Date	HOD/POD	Event or Intervention
2024-02-16		Admission to tertiary hospital with acute cerebral infarction
2024-02-22		Total gastrectomy for acute peptic ulcer with hemorrhage and perforation
2024-03-23	HOD 0	Transfer to our hospital for further critical care
2024-03-25	HOD 2	First EGD (Figure 1A-1C), first CMV RQ-PCR blood test and IV ganciclovir start
2024-03-26	HOD 3	Abdominopelvic CT
2024-03-27	HOD 4	Angioembolization of the jejunal branch
2024-04-08	HOD 16	EVT (Figure 1D-1F)
2024-04-17	HOD 25	Completion of intravenous ganciclovir therapy
2024-04-29	HOD 37	EVT removal and balloon dilation (Figure 1G)
2024-05-16	HOD 54	SEMS insertion (Figure 1H, 1I)
2024-07-16	HOD 115	Death due to deterioration following a presumed aspiration event
Case 2		
Date	HOD/POD	Event or Intervention
2019-01-29		Open low anterior resection for sigmoid colon cancer
2019-03-13 – 2019-11-19		Adjuvant chemotherapy (FOLFOX #12)
2025-02-24	HOD 1, POD 0	Laparoscopic subtotal gastrectomy with Roux-en-Y gastrojejunostomy for gastric cancer
2025-03-05	POD 9	First exploratory laparotomy for perforation in descending colon and 0.8-cm defect jejunojejunostomy (Figure 3A)
2025-03-07	POD 2 after first exploratory laparotomy	Abdominopelvic CT (Figure 3B) and EVT (Supplementary Figure S1A, S1B)
2025-03-23	POD 18 after first exploratory laparotomy	Abdominopelvic CT (Figure 3C) and second exploratory laparotomy
2025-03-30	POD 7 after second exploratory laparotomy	Abdominopelvic CT (Figure 3D)
2025-04-01	POD 9 after second exploratory laparotomy	First CMV RQ-PCR blood test and EVT (Supplementary Figure S1C, S1D)
2025-04-20	POD 28 after second exploratory laparotomy	Completion of intravenous ganciclovir therapy
2025-08-20	HOD 178	Discharge

HOD, hospital day; POD, postoperative day; EGD, esophagogastroduodenoscopy; CMV, cytomegalovirus; RQ-PCR, real-time quantitative polymerase chain reaction; CT, computed tomography; EVT, endoscopic vacuum therapy; SEMS, self-expandable metallic stent.

Supplementary Figure S1. Endoscopic findings of Case 2.

(A, B) Endoscopic views obtained on March 7, 2025, showing discrete ulcerative lesions with yellowish exudate around the esophagojejunostomy site.

(C, D) Endoscopic views from April 1, 2025, demonstrating an esophageal ulcer with polypoid granulation tissue at the anterior wall of the mid esophagus.

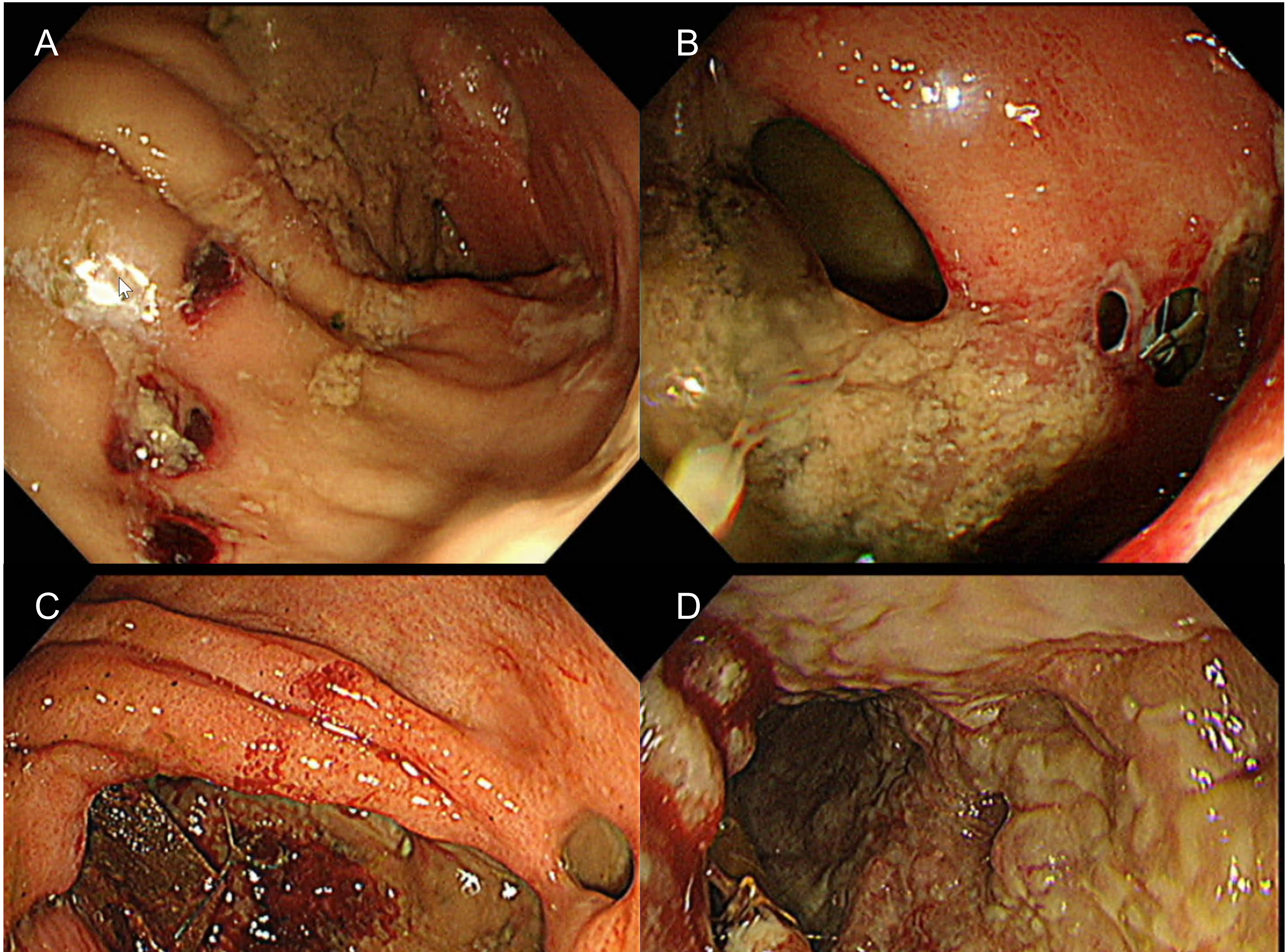

Supplement: Supplementary file 1 [file medi-105-e47254-s001.pdf]
